# Postnatal Imaging of Antenatal Hydronephrosis

**DOI:** 10.1100/tsw.2009.50

**Published:** 2009-05-29

**Authors:** David M. Kitchens, C. D. Anthony Herndon

**Affiliations:** University of Alabama at Birmingham, Birmingham, AL, USA

**Keywords:** pediatrics, hydronephrosis, imaging

## Abstract

Radiologic imaging of the newborn detected prenatally with hydronephrosis should follow a systematic approach. Upper and lower urinary tract imaging should be performed in most cases in order to determine the etiology and gauge the use of future imaging. An overview of renal ultrasound, voiding cystourethrography, renal scintigraphy, and magnetic resonance urography in the setting of antenatal hydronephrosis are discussed.

## POSTNATAL EVALUATION OF ANTENATAL HYDRONEPHROSIS

The postnatal evaluation of the newborn identified with antenatal hydronephrosis (AH) should adhere to a strict protocol that includes a thorough evaluation of both the upper and lower urinary tract (Fig. 1). The differential diagnosis of AH is broad, but ureteropelvic junction (UPJ) obstruction is the most common diagnosis (Fig. 2). A spectrum exists with only a minority requiring surgical intervention. Administration of prophylactic antibiotics is imperative until all imaging studies have been completed. A number of imaging modalities, including renal ultrasound, voiding cystourethrogram, radionuclide renal scan imaging, and magnetic resonance imaging, may be required.

## RENAL/BLADDER ULTRASOUND

Infants are relatively dehydrated at birth, which impacts the recommended timing of the initial renal/bladder ultrasound. In the absence of lower urinary tract obstruction, the initial ultrasound should be performed on the second day of life. Factors such as renal length, anteroposterior renal pelvis diameter, presence of renal cysts, renal parenchymal thickness, and ureteral dilation should be measured. It is important to image the urinary bladder as well as upper urinary system during the assessment. For example, a ureterocele resulting in hydronephrosis may be identified in the bladder. When comparing serial ultrasounds, degree of hydration and status of bladder filling should all be taken into account.

Ultrasound imaging provides adequate anatomic detail in the absence of radiation exposure. However, independently, renal ultrasound is a poor predictor of those patients that will need surgical intervention[1]. In order to standardize evaluations, it is recommended that all images be interpreted with the Society for Fetal Urology (SFU) classification system.

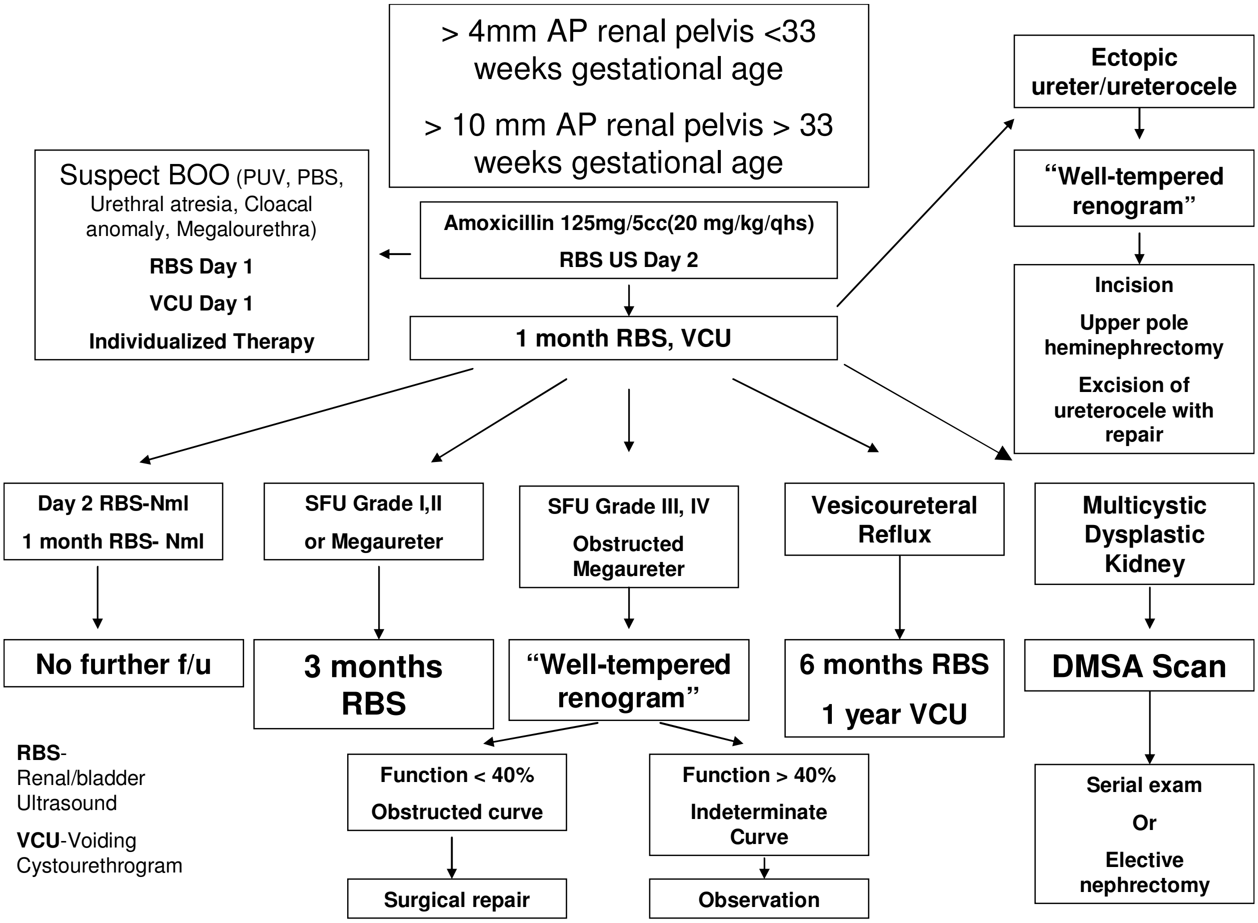

**FIGURE 1.** Postnatal evaluation of AH.

- UPJ obstruction
- Multicystic dysplastic kidney
- Megaureter
- Vesicoureteral reflux
- Ureterocele
- Ectopic ureter
- Ureteral stricture
- Ureteral diverticuli
- Posterior urethral valves
- Prune belly syndrome
- Urethral atresia
- Megacystis-megaureter microcolon
- VACTERL syndrome
- Cloacal anomaly

**FIGURE 2**. Differential diagnosis of AH.

The SFU developed a standardized evaluation system designed in part to help predict patients who may need surgical intervention postnatally[2]. This classification system incorporates collecting system dilation in addition to parenchymal integrity. The system is a spectrum, with grade 1 demonstrating normal parenchymal thickness and only renal pelvis splitting, and grade 4 revealing distention of the renal pelvis and calyces in addition to parenchymal thinning (Fig. 3). Limitations exist with the SFU classification scheme; variables such as renal length in relation to gestational age may improve the predictive value of this system.

| | |
|---|---|
| Grade 1 | Renal pelvis splitting |
| Grade 2 | Moderate renal pelvis splitting confined to renal border |
| Grade 3 | Significant renal pelvis distention outside of renal border, uniform calyceal distention, renal parenchyma normal |
| Grade 4 | Significant renal pelvis distention, significant calyceal distention, renal parenchyma demonstrates thinning |

**FIGURE 3.** SFU grading system for hydronephrosis.

A note of caution. Renal ultrasound protocols vary widely among different centers, which may impact grading and the requirement for additional imaging. For example, at our institution in Birmingham, our radiologists require the patient to be NPO 4 h prior to the image. In contrast, aggressive hydration and preimaging Lasix are given at other institutions (Personal communication with Max Maizels). Without question, this dichotomy will impact significantly not only the grading of the hydronephrosis, but in some cases the requirement for surgical intervention.

## Normal Postnatal Ultrasound

In the presence of a normal postnatal ultrasound, a repeat study is recommended at 1 month of age. If the second study is also normal, in the absence of vesicoureteral reflux, then no further imaging is necessary. The Brussels Free University Perinatal Nephrology Study Group reported a prospective review of patients with mild to moderate AH. In this review, 213 infants (139 boys, 74 girls) with antenatal mild to moderate hydronephrosis were followed at day 5, and months 1, 3, 6, 12, and 24. Eighty-one infants (38%) demonstrated a normal evaluation at birth. In all cases, the exam remained normal throughout the 24-month follow-up period[3]. However, delayed presentations of UPJ obstructions with normal postnatal ultrasounds have been reported and parents should be advised of this[4].

## Abnormal Postnatal Ultrasound

### *Grade 1 or 2 Hydronephrosis*

If the initial ultrasound is abnormal, then a repeat ultrasound is recommended at 1 month. A contemporary standard does not exist for follow-up of SFU grades 1 and 2 hydronephrosis. Also, a controversy exists in the literature concerning the need for long-term follow-up of mild hydronephrosis. Interpretation of the literature is limited by the lack of agreement in terminology of hydronephrosis prior to implementation of the SFU grading system. Some centers report evidence in support of limited follow-up. In 2001, Sairam et al. evaluated the natural history of mid-trimester hydronephrosis. In their review of 268 women, mild (4–10 mm) hydronephrosis was seen in 80% of the fetuses. Of this group, none required surgical intervention postnatally. Furthermore, 88% of this cohort resolved the hydronephrosis in the antenatal or neonatal period[5]. Odibo et al. reported a anteroposterior renal pelvis diameter threshold of 7 mm as predictive of normalization of postnatal studies[6]. In contrast, some reports raise questions to the benign nature of mild pelvic dilation. Looking at the Brussels study of 213 infants with mild to moderate AH, significant uropathies were seen in 39% of the infants[7]. In 1996, Morin et al. reported that surgical intervention for obstruction was required in four out of 63 patients that initially presented with minimal fetal hydronephrosis[8].

Currently, most children with SFU grade 1 and 2 hydronephrosis are followed until they are mature enough to verbalize signs of distress. This age should be individualized. The ideal follow-up protocol for mild forms of hydronephrosis can only be resolved with multicenter prospective trials or registries.

### *Grade 3 or 4 Hydronephrosis*

There is a lack of consensus among pediatric urologists in terms of additional imaging for SFU grade 3 hydronephrosis. Concerns for deterioration in renal function have prompted some to obtain radionuclide scans in all cases of SFU 3 hydronephrosis, while others state that a deterioration does not exist in any case of SFU grade 3 hydronephrosis[9,10]. It should be noted that this dichotomy may stem from the fact that one of these groups performs, in essence, a well-tempered renal ultrasound, thereby potentially up-staging grades of hydronephrosis. This will push some grade 2 to grade 3 and some grade 3 to grade 4 hydronephrosis. Furthermore, Onen has reclassified hydronephrosis in an uncontrolled fashion[10]. These inconsistencies make it virtually impossible to compare similar populations in regards to newborn hydronephrosis and draw meaningful conclusions from their collective observations or meta-analyses. Lee et al. astutely highlighted this fact in the most recent and comprehensive analysis of prenatal hydronephrosis[11]. Nonetheless, this underscores the need to perform serial imaging at the same center in order to allow for consistency in interpretation and recommendations for surgical intervention.

A consensus does exist for the management of grade 4 hydronephrosis. Most centers repeat an ultrasound monthly in addition to obtaining a well-tempered diuretic renogram. Surgical intervention will be required in most of these patients.

### Evaluation of Bilateral Hydronephrosis in a Male

Any male fetus with antenatal bilateral hydronephrosis and a dilated bladder should be presumed to have posterior urethral valves. It is incumbent on the physician to recognize that posterior urethral valves represent a potentially life-threatening condition if a delay in diagnosis is made. Typically SFU grade 3 or 4 hydronephrosis, hydroureter, renal dysplasia, a thickened bladder wall, and low amniotic fluid will exist in the patient with urethral valves, but this is not absolute. The clinician should have a high index of suspicion when evaluating any male with a history of bilateral hydronephrosis. The pediatric urologist should be involved as soon as possible, ideally in the antenatal period.

After delivery, a 5 French feeding tube should be placed in the urinary bladder. A renal ultrasound should be performed on the first day of life and the child placed on prophylactic antibiotics. The voiding cystourethrogram (VCU) should be performed to confirm the diagnosis and child transferred to a tertiary center with the feeding tube decompressing the urinary bladder.

## VOIDING CYSTOURETHROGRAM

A lack of agreement exists concerning the need for a postnatal voiding cystourethrogram in the presence of AH. The overall incidence of vesicoureteral reflux (VUR) in a population with AH ranges from 8 to 38%[12,13,14]. Factors such as the degree of pelvic dilation, race, and the presence of hydronephrosis have been reported to impact the results of the VCU. In favor of a selective approach, Moorthy et al. reported a 1% infection rate in patients evaluated with ultrasound alone for AH[15]. A second study by

Yerkes et al. reported a similar finding in patients with SFU grade 2 hydronephrosis that they followed without performing a postnatal VCU. Intervention was not required in any of the 16 patients[16]. Horowitz et al. reported a significant variance in African-American compared to non-African-American patients and recommended selective screening for the non-African-American cohort[12]. Finally, Ismaili et al. reported that patients with two normal postnatal ultrasounds did not benefit from a VCU[7].

The literature is far more convincing in favor of indiscriminate postnatal VCU for hydronephrosis that is detected prenatally. A number of centers, including one multicenter review, have reported the initial postnatal ultrasound to be normal in up to 25% of patients with VUR that were identified with AH[14,17,18]. Herndon et al.[17] reported a majority of VUR to be high grade and bilateral for infants that demonstrated a normal initial postnatal ultrasound. Dudley et al. reported that all of the patients with antenatally detected VUR demonstrated postnatal hydronephrosis of less than 10 mm. In addition, Herndon et al. reported that 88% of patients with antenatal VUR demonstrated mild hydronephrosis postnatally. Although some controversy exists in the literature, VCU is strongly recommended for the fetus with AH regardless of the initial postnatal ultrasound. Antibiotic prophylaxis should be continued until the VCU is completed.

## RADIONUCLIDE RENAL SCAN

When concerns for obstruction exist, a “well-tempered diuretic diethylene triamine pentaacetic acid (DTPA) or mercaptoacetyltriglycine (MAG-3) renogram” should be performed[19]. This is a dynamic modality that provides relative function and drainage to aid the urologist in assessing the obstructive nature of AH. Of the two available agents, MAG-3 is preferred because of its favorable renal secretion properties. As with all studies, limitations exist and the efficacy of this modality varies significantly in the literature. Several important points deserve mention. First, all patients should have a catheter placed in order to eliminate bladder pressure and prevent reflux of the radionuclide. Second, Lasix administration should be given with an appropriate dose at the point of maximal tracer uptake. Third, intravenous hydration is required in all patients. Despite these guidelines, significant variances have been reported. Some centers have demonstrated a significant alteration in relative function based on the size of the region of interest (ROI). The Great Ormond Street group reported that the relative variability of the ROI in the larger hydronephrotic kidney may explain the common finding of supranormal renal function[20]. In another study, Takla et al. reported on 51 patients with AH. Normalization of renal function and washout was demonstrated in 55%. The initial drainage curve was predictive of those patients that eventually normalized their studies. Normalization of renal drainage and function occurred in 86% of patients with an initial nonobstructive curve, 62% with an indeterminate scan, and 18% with an initial obstructive curve[21]. In contrast, Hafez et al. reported that the initial drainage curve from the initial renogram were not predictive of those patients requiring surgical intervention for UPJ obstruction[1].

The current recommendation for surgical intervention is an obstructive washout curve in which the T ½ exceeds 20 min or a significant discrepancy in split renal function (<40%) is detected. One caveat to split renal function is the patient with severe bilateral hydronephrosis. In this setting, split renal function is not an accurate indictor of overall renal function because of the absence of a normal control to which to compare the hydronephrotic kidney. In this scenario, the renal unit that demonstrates the least function and, more importantly, the worst drainage should undergo repair. Although the indications for surgical intervention may appear straightforward, the drainage curve alone may be significantly altered if the child is dehydrated or furosemide is given too early in the massively hydronephrotic kidney. In addition, the actual renal function may be significantly over-represented in the large hydronephrotic kidney. Based on these inherent weaknesses, we utilize the renal scan to document baseline renal function and as a complement to the renal ultrasound more than an independent predictor of obstruction.

## MAGNETIC RESONANCE IMAGING UROGRAPHY (MRU)

As an imaging modality, MRU offers the advantages of providing superior imaging detail and functional assessment of obstruction without neonatal radiation exposure. MRU is still in its infancy in terms of development and application for prenatal hydronephrosis. A majority of the data for this modality comes from the Emory group. MRU provides excellent anatomic imaging as well as functional determination in the classification of obstructed systems[22,23,24,25]. In 2006, Kirsch et al. presented data that demonstrated an improvement in renal transit time as well as the Patlak score, which is a determinant of single kidney glomerular filtration rate[26]. As a determinant of predicting the need for pyeloplasty, Kaneyama et al. looked at the level of ureteral insertion into the renal pelvis. This group found that a ratio of greater than 0.3 for distance of ureteral insertion to the length of the calyx was predictive of the need for surgical intervention of UPJ obstruction[27].

Unfortunately, the level of scientific evidence in favor of the use of MRU for the evaluation of prenatal hydronephrosis is fairly poor. Studies available for evaluation are uncontrolled. In addition, issues such as cost, availability, and the need for sedation in most patients significantly limit the widespread application of this imaging modality.

## SUMMARY

In summary, postnatal imaging of the infant with a prenatal history of hydronephrosis should be thorough and follow a strict algorithm. Prophylactic antibiotics are imperative until the evaluation is complete. The most common anomalies will be identified early in the evaluation process, leading to an individualized patient and diagnoses-specific treatment plan.
